# A transgenic *Xenopus laevis* reporter model to study lymphangiogenesis

**DOI:** 10.1242/bio.20134739

**Published:** 2013-07-11

**Authors:** Annelii Ny, Wouter Vandevelde, Philipp Hohensinner, Manu Beerens, Ilse Geudens, Antonio Diez-Juan, Katleen Brepoels, Stéphane Plaisance, Paul A. Krieg, Tobias Langenberg, Stefan Vinckier, Aernout Luttun, Peter Carmeliet, Mieke Dewerchin

**Affiliations:** 1 Laboratory of Angiogenesis and Neurovascular link, Vesalius Research Center, VIB, 3000 Leuven, Belgium; 2 Laboratory of Angiogenesis and Neurovascular link, Vesalius Research Center, KU Leuven, 3000 Leuven, Belgium; 3 Center for Molecular and Vascular Biology, KU Leuven, 3000 Leuven, Belgium; 4 Department of Cell Biology and Anatomy, University of Arizona College of Medicine, Tucson, AZ 85724-5044, USA; ‡ Present address: Instituto de Investigación Sanitaria INCLIVA, 46010 Valencia, Spain

**Keywords:** *Xenopus*, Lymphangiogenesis, Imaging

## Abstract

The importance of the blood- and lymph vessels in the transport of essential fluids, gases, macromolecules and cells in vertebrates warrants optimal insight into the regulatory mechanisms underlying their development. Mouse and zebrafish models of lymphatic development are instrumental for gene discovery and gene characterization but are challenging for certain aspects, e.g. no direct accessibility of embryonic stages, or non-straightforward visualization of early lymphatic sprouting, respectively. We previously demonstrated that the *Xenopus* tadpole is a valuable model to study the processes of lymphatic development. However, a fluorescent *Xenopus* reporter directly visualizing the lymph vessels was lacking. Here, we created transgenic *Tg(Flk1:eGFP) Xenopus laevis* reporter lines expressing green fluorescent protein (GFP) in blood- and lymph vessels driven by the Flk1 (VEGFR-2) promoter. We also established a high-resolution fluorescent dye labeling technique selectively and persistently visualizing lymphatic endothelial cells, even in conditions of impaired lymph vessel formation or drainage function upon silencing of lymphangiogenic factors. Next, we applied the model to dynamically document blood and lymphatic sprouting and patterning of the initially avascular tadpole fin. Furthermore, quantifiable models of spontaneous or induced lymphatic sprouting into the tadpole fin were developed for dynamic analysis of loss-of-function and gain-of-function phenotypes using pharmacologic or genetic manipulation. Together with angiography and lymphangiography to assess functionality, *Tg(Flk1:eGFP)* reporter tadpoles readily allowed detailed lymphatic phenotyping of live tadpoles by fluorescence microscopy. The *Tg(Flk1:eGFP)* tadpoles represent a versatile model for functional lymph/angiogenomics and drug screening.

## Introduction

Blood- and lymph vessels are essential for the transport of fluids, gases, macromolecules and cells within vertebrates (Adams and Alitalo, 2007; Alitalo, 2011). Numerous serious disorders, such as cancer, lymphedema, inflammation and diabetic complications are exacerbated or caused by impaired formation or dysfunction of these vasculatures (Alitalo, 2011; Carmeliet and Jain, 2011; Jurisic and Detmar, 2009; Tammela and Alitalo, 2010; Wang and Oliver, 2010). While several anti-angiogenic strategies have been approved or are under clinical trial for the treatment of human malignancies (Carmeliet and Jain, 2011), strategies for the specific modulation of lymphatic growth remain scarce (Alitalo, 2011; Jurisic and Detmar, 2009; Tammela and Alitalo, 2010; Wang and Oliver, 2010). This is in part due to the still limited knowledge of the molecular regulation of lymphatic development, a prerequisite to identify pro- or anti-lymphangiogenic candidates.

Small vertebrate models such as zebrafish and frog (*Xenopus*) embryos have greatly contributed to the molecular deciphering of biological processes, including vascular development (De Smet et al., 2006; Ny et al., 2006; Robert and Cohen, 2011; Wheeler and Brändli, 2009). We previously established the *Xenopus laevis* tadpole as a genetic model for lymphangiogenesis research, phenocopying deficiencies of known mammalian lymphatic genes (Ny et al., 2005). We and others further applied the tadpole model to investigate molecular regulation of lymphatic vascular development, including its use in chemical library screens to identify anti-lymph/angiogenesis compounds (Kälin et al., 2009; Marino et al., 2011; Ny et al., 2008; Leslie Pedrioli et al., 2010). In these studies, visualization of the blood- and lymphatic vasculature depended on staining by in situ hybridization (ISH). Although an excellent tool as such, drawbacks of ISH include its lengthy protocol (days), poor cellular resolution, inappropriateness for dynamic live imaging, and technical difficulties for whole mount ISH staining beyond a certain developmental stage (stage 42, i.e. 4 days postfertilization (dpf)). Fluorescent reporters could circumvent these problems. Indeed, in zebrafish for instance, transgenic lines with fluorescent reporter expression in blood and/or lymphatic endothelial cells have facilitated the identification or characterization of (lymph)angiogenic genes (Bussmann et al., 2010; Cha et al., 2012; Hogan et al., 2009; Küchler et al., 2006; Lawson and Weinstein, 2002; Tao et al., 2011; Yaniv et al., 2006). The *Xenopus* tadpole is a powerful complementary model as it possesses specific advantages over zebrafish embryos (among others the development of a complex and functional lymphatic network within 4 to 5 days of embryonic development and allowing lymphatic commitment, sprouting and migration studies; larger size allowing easier functional lymph/angiography; evolutionary closer to humans). Here, we report the first transgenic *Xenopus laevis* reporter line expressing GFP in both the blood- and lymphatic vasculature under the xFlk1 promoter (*Tg(Flk1:eGFP)*). We validated the model using genetic or pharmacological inhibition, further applied it to phenotype lymphangiogenic processes and established novel in vivo models of spontaneous and induced lymphatic/vascular sprouting, in combination with a labeling method selectively visualizing lymphatics with cellular resolution up to the sprouting lymphatic tip cell.

## Results

### Generation of *Tg(Flk1:eGFP) Xenopus laevis*

We used restriction enzyme mediated integration (REMI) (Kroll and Amaya, 1996) to generate transgenic *Xenopus laevis* reporter tadpoles with vascular endothelial GFP expression. The *Xenopus laevis* Flk1 (VEGFR-2) promoter and first intron were used to drive reporter expression (supplementary material Fig. S1). At stage 45, tadpoles were screened for vascular GFP signal and positive tadpoles were raised, yielding 14 GFP^+^ F0 animals that survived to adulthood. Nine of these (all males) were crossed with wild type females to determine germline transmission of the transgene and to profile GFP expression in the F1 offspring (supplementary material Table S1). Five of the founders sired offspring with strong and exclusive expression in the vasculature. All further experiments were performed using offspring from these founders, collectively referred to as *Tg(Flk1:eGFP)* lines.

### *Tg(Flk1:eGFP)* tadpoles express GFP in blood and lymph vessels

**Fig. 1.**
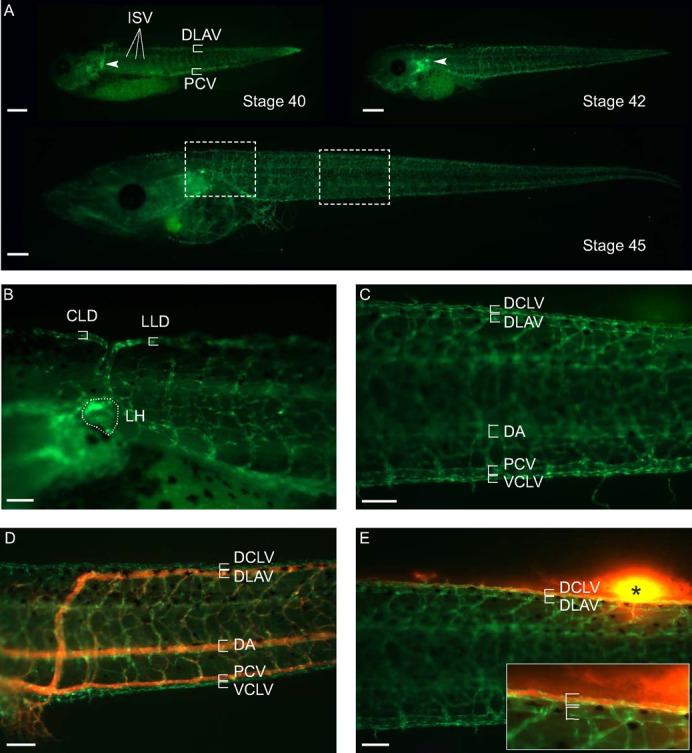
GFP expression in blood and lymphatic vessels of *Tg(Flk1:eGFP)* tadpoles. All panels depict lateral views of the tadpoles, head facing left. (**A**) Stage 40–45 *Tg(Flk1:eGFP)* tadpole showing GFP signal in the entire blood and lymphatic vasculature. (**B**) Higher magnification of fluorescent LH (encircled) and connecting lymphatic vessels in a stage 45 tadpole. The region demarcated by the left square in panel A is shown. (**C**) Higher magnification of GFP^+^ blood and lymphatic vessels in the trunk of a stage 45 tadpole. The region demarcated by the right square in panel A is shown. (**D**) Angiography by intracardial injection of high molecular TRITC-dextran exclusively labeled the blood vasculature (shown in orange). (**E**) Reversely, lymphangiography by injecting the TRITC-dextran dye in the fin adjacent to the DCLV (black asterisk denotes site of injection), showing specific uptake of the dye by the lymphatics (shown in orange) and draining towards the lymph heart. Inset shows higher magnification of the zone proximal to the injection site. CLD, cephalic lymph duct; DA, dorsal aorta; DCLV, dorsal caudal lymph vessel; DLAV, dorsal longitudinal anastomosing vessel; ISV, intersomitic vessel; LH, lymph heart; LLD, lateral lymph duct; PCV, posterior cardinal vein; VCLV, ventral caudal lymphatic vessel. Scale bars: 500 µm (A), 200 µm (B–E).

To characterize transgenic GFP expression in the vasculature, F1 tadpoles were monitored by fluorescent microscopy. Upon gradual disappearance of autofluorescent signal in the embryo and yolk, the first identifiable GFP^+^ vascular structures were the posterial cardinal vein (PCV) and intersomitic vessels (ISVs) at stage 35–36 (2.5 dpf) (not shown). The spatio-temporal onset of the fluorescent signal corresponded to the VEGFR-2 expression pattern as demonstrated by in situ hybridization (Cleaver et al., 1997). At stage 40 and beyond, when the autofluorescent yolk was further retracted and the tadpoles became more transparent, the GFP signal was detectable in the vascular network in the tail and the head, and was present in the entire blood vasculature by stage 45 (Fig. 1A). In all *Tg(Flk1:eGFP)* tadpoles, GFP was also expressed in the lymphatic vasculature. Close examination at stage 40–45 (3–5 dpf) revealed that the developing lymph heart was GFP^+^ (Fig. 1A,B). In addition, the connecting lymph vessels, such as the cephalic lymph duct (CLD) and the lateral lymph duct (LLD) (Ny et al., 2005) were GFP^+^ (Fig. 1B), as well as the large axial lymph vessels, the ventral caudal lymph vessel (VCLV) ventrally of the PCV, and the dorsal caudal lymph vessel (DCLV) dorsally of the dorsal longitudinal anastomosing vessel (DLAV) (Fig. 1C).

The identity of the GFP^+^ vessels was further confirmed using functional assays that specifically double label blood- versus lymph vessels. Upon angiography by intracardial micro-injection of a red fluorescent dye (tetramethylrhodamine-dextran, TRITC-dextran, Mr 2×10^6^ Da), the otherwise green blood vessels in *Tg(Flk1:eGFP)* tadpoles became orange, while the VCLV and DCLV remained green (Fig. 1D). Conversely, following lymphangiography by subcutaneous injection of TRITC-dextran in developing tadpoles, lymph vessels in *Tg(Flk1:eGFP)* tadpoles became orange, while blood vessels remained green (Fig. 1E). Thus, both blood- and lymph vessels can be readily visualized in the *Tg(Flk1:eGFP)* tadpoles by their fluorescence.

### A novel method for selective and prolonged labeling of lymph vessels

**Fig. 2.**
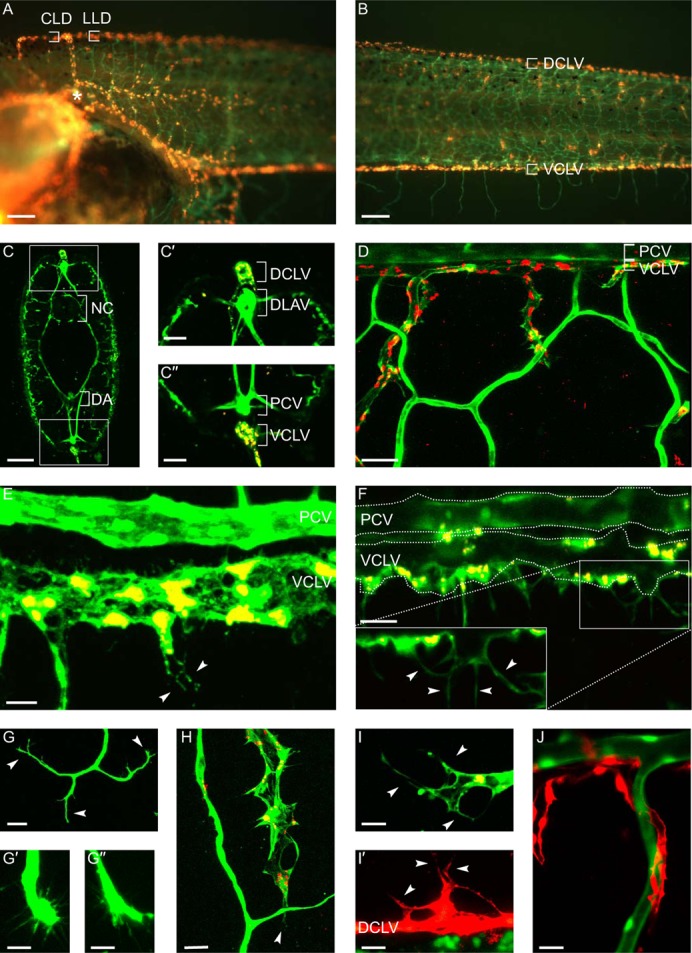
In vivo labeling of lymphendothelial cells allows parallel blood- and lymph vessel analysis. (**A**,**B**) Specific “LEC labeling” of lymph vessels (orange) by TRITC-dextran 24 hours after angiography of stage 46 tadpoles, allowing prolonged visualization of the vessels in the lymph heart (asterisk) area (A) and tail (B). (**C–C″**) Confocal imaging of cross sections of a LEC labeled tadpole confirmed exclusive labeling of lymphatics (DCLV, VCLV), while blood vessels (PCV, DA) remain green. C′–C″ are higher magnifications of the dorsal (C′) and ventral (C″) frames in panel C. (**D**) High magnification of vascular sprouting into the fin showed distinct morphology between smooth blood vessels (green) and the spiky lymphatics (red/orange). (**E**,**F**) Blood vessel (PCV, green) and lymph vessel (VCLV, yellow/organge) in close proximity. LECs of the VCLV exhibit small protrusions and filopodia (arrowheads). (**G–G″**) Newly forming blood vessel sprouts display a typical tip cell phenotype (arrowheads) with several filopodia. G′–G″ are higher magnifications of angiogenic tip cells. (**H**) Lymphatic tip cell with protruding filopodia (arrowhead). (**I**,**I′**) High magnification of lymphatic tip cells with filopodia (arrowheads) at the forefront of a lymph vessel in the fin (I) or of a new sprout forming from the DCLV (I′). (**J**) Lymphatics (red) occasionally sprouted at the same site as blood vessels (green), seemingly using them as a scaffold for further elongation into the fin. CLD, cephalic lymph duct; DA, dorsal aorta; DCLV, dorsal caudal lymph vessel; LLD, lateral lymph duct; NC, notochord; PCV, posterior cardinal vein; VCLV, ventral caudal lymph vessel. Scale bars: 200 µm (A,B), 100 µm (C), 50 µm (C′,C″,D,G), 20 µm (E,F,H,I′,J), 10 µm (G′,G″,I).

We also established an additional method to selectively label lymphatic endothelial cells (LECs) for protracted periods. This was achieved by intracardial injection of TRITC-dextran, initially labeling only the blood vessels, but allowing the dye to extravasate into the interstitial space. After 24 hours, the dye is then taken up by the LECs, likely by a process of pinocytosis, and was stably retained inside the LECs as well as their daughter cells for prolonged times (≥2 weeks). This LEC labeling method selectively labeled lymphatic structures such as the lymph hearts, head lymphatics, axial lymphatics of the tail (Fig. 2A,B), as further confirmed by confocal imaging of cross sections (Fig. 2C–C″). qRT-PCR analysis of FACS sorted GFP^+^ blood vascular ECs (BECs) and GFP^+^TRITC-dextran^+^ LECs revealed higher levels of the LEC marker genes *Prox1, VEGFR-3, LYVE-1* and *reelin* in the LECs, confirming their lymphatic lineage (supplementary material Fig. S2). Thus, the LEC labeling method allowed color distinction between blood and lymph vessels in a fast and easy manner and for prolonged times, enabling dynamic monitoring of vascular development in a single tadpole. Of note, LEC labeling was also possible in conditions where lymphatic vessels were malformed and dysfunctional (see below).

### Documentation of lymphangiogenic sprouting and patterning in the fin

Follow-up of vascular development in older stage embryos revealed that lymph vessels branched off into the fin by sprouting, just like axial blood vessels did (Rhodin and Lametschwandtner, 1993). As the fin is originally devoid of lymphatics, and lymphangiogenesis into the fin was not described to date, we documented day-by-day for 14 days the sequence and pattern of fin vascularization; to distinguish lymphatic from blood vessel sprouts by color, LECs were labeled by TRITC-dextran (supplementary material Fig. S3). Blood vessel sprouting preceded lymphatic sprouting and first occurred in the ventral fin starting from stage 46 onwards (6 dpf; supplementary material Fig. S3A′). Blood vessel sprouting was not stereotyped but occurred in a random pattern, however the final sprout number was comparable between tadpoles (not shown). Blood vessel sprouts then turned back towards the axial vessels forming closed loops (supplementary material Fig. S3B,B′). Later on, existing loops developed secondary angiogenic branches, forming a network covering the entire fin. Lymph vessel sprouting started only from stage 48 (8 dpf) onwards. Lymphatics sprouted from the VCLV and DCLV without evidence of a stereotyped spatio-temporal pattern of branch formation. Compared to blood vessels, fewer lymphatic sprouts developed, in turn forming only a few secondary branches that at a later stage interconnected to form the mature fin lymphatic network (supplementary material Fig. S3B–D′).

Confocal microscopy showed that blood vessels in the fin had a smooth contour and shape (Fig. 2D), while lymph vessels appeared more spiky (Fig. 2D,H,J). Furthermore, the axial lymphatics in the tadpole body displayed LECs with many small protrusions, a feature that was not observed in axial blood vessels (Fig. 2E,F). High-magnification confocal imaging and time-lapse video-recording showed that the leading cells of both blood and lymphatic sprouts displayed a typical “tip cell” phenotype, with several filopodia-like structures to sense the environment (Fig. 2E,G–I′; supplementary material Movie 1). Occasionally, lymph vessels sprouted close to blood vessel sprouts, possibly using the latter as a scaffold for further elongation into the fin (Fig. 2J).

### *Tg(Flk1:eGFP)* tadpoles as a tool for functional lymphangiogenomics

**Fig. 3.**
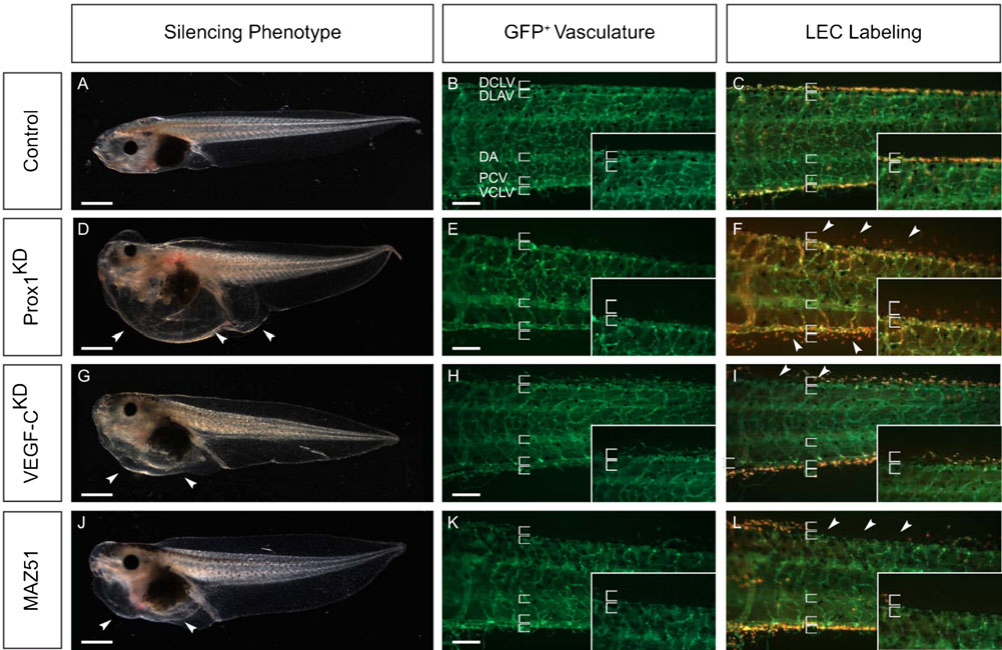
*Tg(Flk1:eGFP)* transgenic tadpoles as a tool to study developmental lymphangiogenesis. All panels depict lateral views of stage 45 tadpoles, head facing left. All insets show higher magnification of the dorsal side, with top and lower bracket denoting DCLV and DLAV, respectively. (**A–C**) Control embryo showing normal morphology (A) and correctly formed GFP^+^ (B) and LEC labeled lymphatics (C). (**D–F**) Morpholino knockdown (KD) of xProx1 resulted in edema around heart, gut and cloaca (arrowheads in panel D). GFP fluorescent microscopy showing that Prox1^KD^ tadpoles possess few and disorganized LECs failing to assemble into the dorsal and ventral lymphatics (E). LEC labeling showing fewer LECs in xProx1^KD^ tadpoles. Arrowheads denote sites where longitudinal lymph vessels are missing or malformed (green: DLAV, orange: DCLV) (F). (**G–I**) Morpholino knockdown (KD) of xVEGF-C resulted in edema in the heart and gut (arrowheads in panel G). Reporter VEGF-C^KD^ tadpoles revealing a fragmented DCLV consisting of dispersed and scattered LECs on the dorsal side while the VCLV appears grossly normal as shown by fluorescent microscopy (H) and LEC labeling (I). Arrowheads denote sites where longitudinal lymph vessel is missing. (**J–L**) Chemical inhibition of VEGFR-3 by MAZ51 treatment (10 µM) resulted in edema in the heart and gut (arrowheads in panel J). Fluorescent microscopy (K) and LEC labeling (L) revealing a disorganized and fragmented DCLV consisting of few LECs, while the VCLV appeared normal. Arrowheads denote where longitudinal lymph vessels are missing. DA, dorsal aorta; DCLV, dorsal caudal lymph vessel; DLAV, dorsal longitudinal anastomosing vessel; PCV, posterior cardinal vein; VCLV, ventral caudal lymph vessel. Scale bars: 1 mm (A,D,G,J), 250 µm (B,E,H,K).

To validate the *Tg(Flk1:eGFP)* line for lymphangiogenesis research, we first performed morpholino oligomer-mediated silencing of two known lymphangiogenic genes, *Prox1* and *VEGF-C*, previously shown to cause severe lymphatic defects in non-reporter tadpoles (Ny et al., 2005; Ny et al., 2008). All tadpoles injected with control morpholino developed normally with well-structured and functional blood vessels and lymphatics as readily visualized by fluorescent stereomicroscopy in combination with LEC labeling (Fig. 3A–C). In contrast, knockdown of xProx1 in *Tg(Flk1:eGFP)* tadpoles resulted in edema formation at stage 45/46 (5–6 dpf) (Fig. 3D) and dysfunctional lymphatics at an incidence similar to what we previously observed in non-reporter tadpoles (Ny et al., 2005) (not shown). Fluorescent microscopy for GFP showed that both the VCLV and the DCLV were severely compromised and hardly detectable (Fig. 3E). LEC labeling further revealed a highly disorganized and dilated appearance with few and loosely attached LECs (Fig. 3F). As observed in non-reporter tadpoles (Ny et al., 2005), lymphatic defects were more severe for the DCLV (often completely absent) than for the VCLV (Fig. 3E,F). Likewise, silencing of xVEGF-C in *Tg(Flk1:eGFP)* tadpoles caused edema (Fig. 3G) and severe lymph vessel anomalies (fewer and more dispersed and scattered LECs) which were readily visualized by fluorescence microscopy (Fig. 3G–I). These results are in accordance with previous observations in VEGF-C deficient mice and in VEGF-C silenced non-reporter tadpoles (Karkkainen et al., 2004; Ny et al., 2005; Ny et al., 2008). Thus, morphant phenotypes in *Tg(Flk1:eGFP)* and non-reporter tadpoles were comparable (Ny et al., 2005). However, the *Tg(Flk1:eGFP)* model enabled superior imaging of vascular morphology and function for both blood and lymph vessels, and in a dynamic manner in live tadpoles.

### *Tg(Flk1:eGFP)* tadpoles as a tool for chemicogenetics

We also evaluated the *Tg(Flk1:eGFP)* line to facilitate screening of anti-lymphangiogenic chemical compounds. As proof-of-principle, we used MAZ51, a chemical inhibitor with a >10-fold higher selectivity for VEGFR-3, the receptor for the lymphangiogenic factors VEGF-C and VEGF-D (Tammela and Alitalo, 2010) than for the other (angiogenic) VEGF receptors (Kirkin et al., 2001). We previously documented the lymphatic phenotype of MAZ51 in non-reporter tadpoles (Ny et al., 2008). To minimize any adverse effect of MAZ51 on general development, we exposed tadpoles to this compound only beyond stage 26/28, i.e. just prior to the formation of the major blood and lymph vessels (Levine et al., 2003; Ny et al., 2005), and at a concentration (10 µM) that affected the blood vasculature only minimally (Ny et al., 2008). *Tg(Flk1:eGFP)* tadpoles treated with MAZ51 displayed edema around the heart and gut (Fig. 3J). Microscopic inspection of LEC labeled tadpoles revealed a fragmented DCLV with dispersed and scattered LECs (Fig. 3K,L). Lymphangiography confirmed that the DCLV was dysfunctional (not shown), in accordance with previous observations (Ny et al., 2008). Thus, *Tg(Flk1:eGFP)* tadpoles are useful models for screening compounds targeting the lymphatic (or blood) vasculature.

### Molecular analysis of lymphatic sprouting using the fin assay

**Fig. 4.**
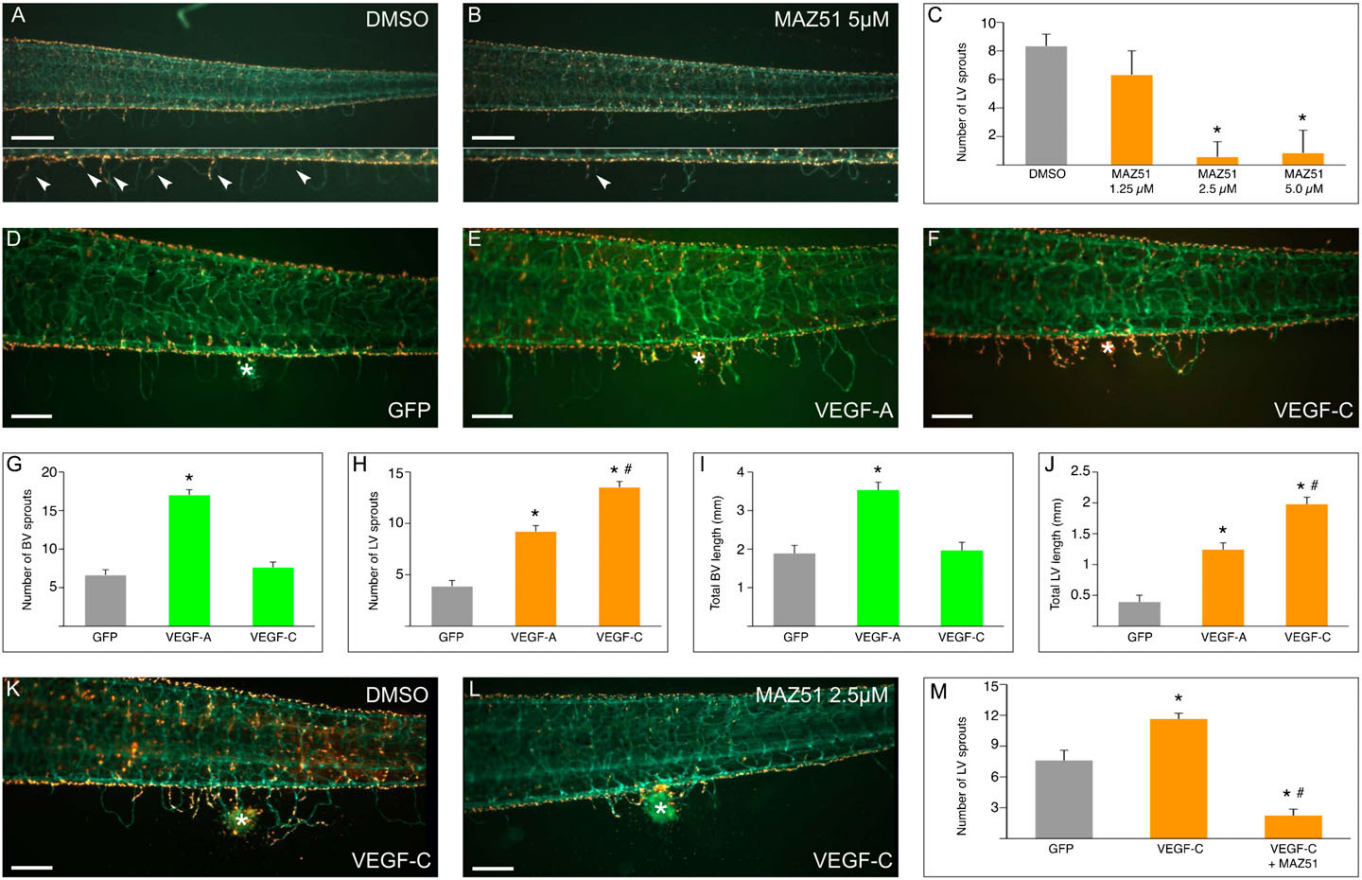
Modulation of lymphatic sprouting. All photos depict lateral views of LEC labeled stage 49 *Tg(Flk1:eGFP)* tadpoles (blood vessels green, lymph vessels orange), head facing left. (**A–C**) Representative micrographs showing inhibition of lymphatic sprouting into the fin (arrowheads) upon MAZ51 treatment (5 µM) (B), as compared to DMSO treated controls (A). Quantification of lymphatic sprout numbers in panel C (*P<0.001 compared to DMSO control). (**D–J**) Representative micrographs of effect of GFP (control) (D), VEGF-A (E) and VEGF-C (F) overexpression on blood and lymph vessel sprouting after injection of transfected cells in the fin. Asterisk denotes the location of the cell cluster. Quantification of the number of blood vessel sprouts (G), lymph vessel sprouts (H), total blood vessel length (I) and total lymph vessel length (J) (*P<0.001 compared to GFP control, #*P*<0.001 compared to VEGF-A overexpression). (**K–M**) Chemical inhibition of VEGF-C induced lymphatic sprouting by MAZ51. Representative micrographs of tadpoles injected with VEGF-C overexpressing cells and treated with DMSO (K) or MAZ51 (L). Corresponding control with GFP overexpressing cells (quantified in panel M) is not shown. Asterisk denotes the location of the cell cluster. Quantification of the number of lymphatic sprouts after MAZ51 treatment in panel M (*P<0.001 compared to GFP control, #*P*<0.001 compared to VEGF-C overexpression). Scale bars: 500 µm (A,B), 250 µm (D–F,K,L). Quantitative data are mean ± s.e.m.

Next, we evaluated whether de novo sprouting of axial vessels into the fin, as documented above, could be exploited as a model to molecularly dissect blood and lymphatic sprouting. Therefore, we used both loss-of-function (LOF) and gain-of-function (GOF) strategies to interfere with angiogenesis and lymphangiogenesis. In the LOF strategy, we screened inhibitory chemical compounds and as a test-case treated tadpoles with increasing concentrations of the VEGFR-3-inhibitor MAZ51 from stage 47 (7 dpf) onwards (Fig. 4A–C). LECs in tadpoles were labeled 24 hours prior to treatment initiation for color distinction between lymphatic and blood vascular sprouts, and were analyzed after one week of treatment. Lymphatic sprouting was inhibited by up to 93±13% (*n* = 15–37, *P*<0.001) when tadpoles were treated with MAZ51 concentrations above 1.25 µM (Fig. 4C). Of note, blood vessel sprouting was only inhibited when MAZ51 was used at 5 µM or higher concentrations (not shown).

In the GOF approach, we developed a procedure to locally overexpress lymph/angiogenic factors in the fin. The fin comprises two epithelial layers surrounding fibroblasts and connective tissue fibers (Rhodin and Lametschwandtner, 1993), into which vascular sprouting occurs. We exploited this micro-environment by injecting *Xenopus* kidney epithelial cells transfected to overexpress vascular growth factors. As proof-of-principle, we tested the model using overexpression of GFP (control), murine VEGF-A or murine VEGF-C. The transfected cells produced detectable levels of the respective protein in the culture supernatant as determined by ELISA (not shown). After injection of a standard volume into the ventral fin, these cells formed a local cluster that persisted for up to a week (supplementary material Fig. S4). Overexpression of VEGF-A, a prominent angiogenic and lymphangiogenic factor (Carmeliet et al., 1996; Nagy et al., 2002), caused pronounced induction of blood and lymph vessels at 4 days after injection. The number and cumulative length of blood vessel sprouts increased by 156±10% and 87±11% over GFP control, respectively, while the number and cumulative length of lymphatic sprouts increased by 137±14% and 217±28%, respectively (*n* = 50, *P*<0.001) (Fig. 4D,E,G–J). Overexpression of the lymphangiogenic growth factor VEGF-C increased lymphatic sprouting, as the number and cumulative length of lymphatic sprouts increased by 247±15% and 406±29%, respectively (*n* = 50, *P*<0.001) (Fig. 4F,H,J). Of note, this increase was significantly higher than that induced by VEGF-A overexpression (*P*<0.001, Fig. 4H,J). VEGF-C overexpression did, however, not affect blood vessel sprouting (*P* = NS versus GFP control; Fig. 4G,I). Next, we evaluated if the effects of VEGF-C on lymphatic sprouting could be reversed by compound inhibitor treatment. Indeed, treatment with 2.5 µM MAZ51 inhibited the VEGF-C-induced increase in lymphatic sprouts by 81±5.4% (*n* = 30–37, *P*<0.001; Fig. 4M) and cumulative length by 87±5.7% (1,713±87 µm for VEGF-C versus 219±97 µm for VEGF-C/MAZ51; *n* = 30–37, *P*<0.001). Thus, two new methods based on vascular sprouting into the fin were established, suitable to genetically dissect lymph/angiogenic sprouting or to screen for modulating chemical compounds.

## Discussion

*Xenopus* and zebrafish embryos have emerged as powerful small animal models for vascular research (Karpanen and Schulte-Merker, 2011; Küchler et al., 2006; Ny et al., 2006; Tammela and Alitalo, 2010; Wheeler and Brändli, 2009). While sharing several features, *Xenopus* possesses specific advantages, including higher genomic and developmental similarity to mammals, and might therefore constitute a particularly useful model for the study of certain vascular aspects (Isogai et al., 2001; Levine et al., 2003; Ny et al., 2005). We previously demonstrated that the *Xenopus* tadpole is a valuable model to study lymphatic development (Ny et al., 2005). While lymph/angiogenesis research benefitted from the use of transgenic vascular reporter zebrafish (Bussmann et al., 2010; Cha et al., 2012; Hogan et al., 2009; Küchler et al., 2006; Lawson and Weinstein, 2002; Yaniv et al., 2006), no such transgenics were available in *Xenopus*. We therefore generated a *Xenopus laevis* reporter line with GFP expression in the blood- and lymphatic vasculature. This was achieved by REMI transgenesis (Kroll and Amaya, 1996) using a *Xenopus laevis* Flk1 promoter/enhancer construct (Meadows et al., 2009), yielding *Tg(Flk1:eGFP)* frogs and tadpoles.

As confirmed by fluorescent microscopy and functional dye injection assays (angiography and lymphangiography), *Tg(Flk1:eGFP)* tadpoles express GFP in both blood and lymph vessels. At the early stages when LEC commitment and migration is initiated (around stage 30–35), the tadpoles are still only semi-transparant and more difficult to image. Shortly after that however, when the tadpoles have become more fully transparent, reporter signal is easily monitored. Previously, a transgenic *Xenopus laevis* reporter line was generated using a similar xflk-1:GFP construct, but no reporter expression was described in the lymphatics (Doherty et al., 2007). Possibly, the use of slightly different promoter or intron fragments yielded BEC-restricted versus our BEC/LEC GFP expression.

We also established a method to fluorescently label the developing lymphatic network in vivo, which enabled easy and persistent color distinction of the two vasculatures in the *Tg(Flk1:eGFP)* lines. Twenty-four hours following a single intra-cardiac injection of fluorescent TRITC-dextran, the LECs of developing lymph vessels have taken up the dye, often retained in vesicle-like structures and appearing as red dots within the cell. Likely, micropinocytotic vesicles, abundant in LEC cytoplasm and participating in normal lymph formation, have taken up the dye via pinocytosis (Ji, 2005). The dye is stably retained for at least 2 weeks, and new lymphatics sprouting from existing labeled ones are automatically also labeled. We observed a variable degree of labeling of LECs between tadpoles, likely due to slight differences in amount of injected dye and explaining why in some images the double-labeled lymphatics appear yellow–orange while red in others (e.g. Fig. 2E,H,I, versus Fig. 2D,I′,J). The expression of LEC specific marker genes by FACS sorted double-labeled GFP^+^TRITC^+^ endothelial cells confirmed their lymphatic identity. Sorted GFP^+^TRITC^−^ BEC and GFP^+^TRITC^+^ LEC populations from the *Tg(Flk1:eGFP*) tadpoles combined with expression profiling may provide a useful tool to screen for novel lymph/angiogenic genes and study differential gene expression in vivo.

We combined reporter expression and LEC labeling to visualize and document in detail the sprouting and patterning of lymphatics from the axial lymph vessels into the fin. High magnification imaging and time-lapse video-recording revealed lymphatic tips cells with several filopodia sensing the environment. Occasionally, lymphatic sprouting occurred in close proximity of the blood vessel sprouts, suggesting that common initiating or guiding cues may be involved.

We validated the *Tg(Flk1:eGFP)* tadpoles as a model for molecular lymphangiogenesis studies, using gene knockdown or compound inhibition of factors critically involved in lymphatic development (Prox1, VEGF-C, VEGFR-3). The phenotypes in the *Tg(Flk1:eGFP)* line and in non-reporter tadpoles (Ny et al., 2005; Ny et al., 2008) were comparable, demonstrating the accuracy and integrity of the model. The added value of the *Tg(Flk1:eGFP*) line for lymph/angiogenesis phenotyping was exemplified by i) the significantly faster procedures as it only requires fluorescent microscopy as compared to lengthy whole mount ISH staining protocols; ii) the unique possibility to analyze defects at developmental stages (>stage 42) non-permissive to whole mount ISH; iii) the significantly higher imaging resolution, enabling analysis of more subtle defects at the cellular level. For instance, the abnormalities of the DCLV/VCLV upon Prox1, VEGF-C or VEGFR-3 blockage could now be seen at a cellular level in the *Tg(Flk1:eGFP)* line, revealing dilated, interrupted vessel-like trunks consisting of sparse and loosely attached LECs. These details were unnoticeable in the counterpart non-reporter morphant tadpoles (Ny et al., 2005; Ny et al., 2008). Recently, the *Tg(Flk1:eGFP)* tadpoles have helped in elucidating the role of novel candidate genes in lymphatic development (Aranguren et al., 2011; Fu et al., 2008; Hermans et al., 2010; Norrmén et al., 2010; Saharinen et al., 2010). These advantages of the model, together with the possibility of dynamic live imaging, and of FACS-sorting of BEC and LEC populations for gene profiling and expression modulation studies, represent significant assets.

We further explored the potential of the de novo vascular sprouting into the fin as an in vivo physiological sprouting model. To that end, we established loss-of-function (LOF) and gain-of-function (GOF) strategies to interfere with sprout formation. LOF experiments demonstrated blockage of de novo sprouting of lymph vessels in the fin by the VEGFR-3 inhibitor MAZ51, exposed to 7-day-old tadpoles. Higher inhibitor concentrations also inhibited blood vessel sprouting. In a second strategy, we explored GOF approaches to modulate fin sprouting. In vivo electroporation with expression plasmids or adenoviral overexpression of known lymphangiogenic factors, while possible in the tail, were not successful in the fin, probably due to its scarce cell content. Implantation of beads coated with recombinant lymphangiogenic proteins was technically challenging, as the fin is very thin and delicate. In contrast, implantation of a dense suspension of *Xenopus laevis* renal cells overexpressing murine lymph/angiogenic factors (VEGF-A or VEGF-C), worked successfully. The transfected cells persisted as a local cluster and induced sprouting of blood and/or lymph vessels. These data also demonstrate that murine VEGF-A and VEGF-C are active in frog with the same specificity toward blood and/or lymphatic endothelial cells as in mouse and humans. Quantification of sprouting (inhibition) in the LEC labeled *Tg(Flk1:eGFP)* tadpoles in both the GOF and LOF model required no more than mere fluorescent microscopy. Sprouting lymphangiogenesis models during embryogenesis in vivo in other species demand either tissue dissection/fixation and staining like in the mouse, or high-resolution confocal/multiphoton imaging such as in the zebrafish embryo (Cha et al., 2012; Geudens et al., 2010; Hogan et al., 2009; Okuda et al., 2012; Tao et al., 2011), and are thus more challenging for screening purposes. The in vivo sprouting models in the tadpole fin are suited to evaluate candidate chemical inhibitors or overexpressed candidate modulating factors in an easy and quantifiable manner, and enable dynamic monitoring of sprouting over several days. In addition, the cell cluster-based fin sprouting assay allows screening for chemical inhibitors for a specific target protein, extending the versatility of the model. Furthermore, whereas the fin sprouting model spans later embryonic stages (up to 14 dpf) and therefore is less compatible with morpholino knockdown approaches, it will also be applicable in future knockout experiments, given the promising recent successes of zinc finger nuclease or TALEn knockout in aquatic species including *Xenopus* (Cade et al., 2012; Lei et al., 2012; Moore et al., 2012; Young et al., 2011).

We conclude that the *Tg(Flk1:eGFP)* reporter line and the assays and models established and validated in this study, provide powerful tools for functional lymphangiogenomics as well as for drug screening, with easy detection, morphological and quantitative characterization of lymphatic phenotypes with resolution up to tip cell and filopodia level.

## Materials and Methods

### Generation of transgenic *Tg(Flk1:eGFP)* reporter tadpoles

The transgenic construct used to generate the *Tg(Flk1:eGFP)* reporter tadpoles was engineered from the *Xenopus laevis* (xl) VEGFR-2/Flk1 gene. Therefore, a genomic fragment containing ∼2.5 kb of promoter sequence up to exon 2, including the complete intron 1 containing enhancer elements essential for the correct expression pattern, was isolated and cloned into pBSK(+) (Meadows et al., 2009). Next, the eGFP cDNA fused to the SV40 polyA was inserted into exon 1 to drive reporter expression under the control of the xlFlk1 promoter. The construct was linearized using KpnI and used in restriction enzyme mediated integration (REMI) transgenesis (Kroll and Amaya, 1996) using purified sperm nuclei and unfertilized eggs from wildtype frogs purchased from Nasco Biology (Fort Atkinson, WI). Transgenic tadpoles were screened for eGFP signal in the vasculature at stage 45 by fluorescence microscopy and positive tadpoles were raised to adulthood. F1 tadpoles were generated by natural breeding of *Tg(Flk1:eGFP)* F0 males with wild type females; F2 tadpoles by interbreeding male and female *Tg(Flk1:eGFP)* F1 frogs. All breeding was done using hormonal induction. All animal procedures were approved by the ethical committee for animal experimentation of the KU Leuven, Leuven, Belgium.

### In vivo imaging of blood- and lymphatic vasculature in *Tg(Flk1:eGFP)* tadpoles

Stage 45–46 tadpoles were anesthetized in 0.02% 3-aminobenzoic acid ethyl ester, and placed on agarose gel. Tetramethylrhodamine-dextran (TRITC-dextran, Mr 2×10^6^, Invitrogen, Merelbeke, Belgium) dye was injected close to the dorsal or ventral caudal lymph vessel (DCLV or VCLV, respectively) or in the heart with glass capillaries using a micromanipulator and a Zeiss SV11 stereomicroscope to monitor lymphatic function (lymphangiography) and to label functional blood vasculature (angiography), respectively. Lymphendothelial cell (LEC) labeling was performed by injecting 10–20 nl of TRITC-dextran in or around the heart of anesthetized tadpoles. After 24 hours, the injected dye is taken up inside the LECs of the lymphatic vasculature. Fluorescent images (of anesthetized tadpoles) were acquired with the Zeiss AxioVision 4.6 software on a Zeiss Lumar V.12 fluorescence stereomicroscope equipped with a Zeiss AxioCam MrC5 digital camera (Zeiss, Zaventem, Belgium). Confocal images of blood- and lymph vessel sprouts and tip cells were obtained from anesthetized and agarose embedded (1% low gelling agarose) tadpoles with a Zeiss CLSM510 NLO META mounted on an AxioVert200M (Zeiss, Zaventem, Belgium) inverted microscope. Confocal imaging of GFP was performed using 920 nm pulsed mode-locked laser emission from a tunable Ti:Sapphire Chameleon laser (Coherent, Utrecht, The Netherlands); TRITC-dextran imaging was performed using a DPSS 561-10 laser. Time-lapse imaging (2 hours) was performed with minimal necessary laser power. Stacks of frame-averaged (four frames) confocal optical slices were collected digitally, at 5 minute intervals for time-lapse sequences. Three-dimensional as well as four-dimensional reconstructions of image data were prepared using LSM software package.

### FACS sorting of BECs and LECs

Five-day-old tadpoles were injected with 10 nl TRITC dextran into the heart sac and further processed 24 hours later to obtain single cell suspensions. Briefly, tadpoles were bleached for 5 minutes, rinsed with distilled water, macerated, and incubated in 0.25% trypsin at 28°C until almost fully digested. A couple of drops of fetal bovine serum (FBS) were added to inhibit the trypsin activity and the digest was diluted with PBS to approximately 25 ml before filtering over a 70 µm nylon mesh (BD Biosciences, Erembodegem-Aalst, Belgium). Cells were pelleted by centrifugation at 600 g for 7 minutes and the cell pellet was resuspended in 4 ml PBS containing 1% bovine serum albumin (BSA). Cells were sorted on a FACSAria (BD Biosciences, Erembodegem-Aalst, Belgium), taking care to exclude possible doublets or cell clusters. Non-injected GFP^+^ and TRITC-injected GFP^−^ tadpoles were used as controls for proper compensation and gate setting. On average 25,000 GFP^+^TRITC^+^ LECs and 50,000 GFP^+^TRITC^−^ blood endothelial cells (BECs) were sorted directly in lysis buffer (RLT containing 1% β-mercaptoethanol; Qiagen, Venlo, The Netherlands) from 60–100 pooled tadpoles. RNA was prepared from these samples using the QIAGEN mini kit and cDNA was prepared using the SuperscriptIII kit (Ambion, Lennik, Belgium). Gene expression was analyzed by qRT-PCR using SYBR Green (Applied Biosystems, Lennik, Belgium) and home-designed primer sets (prox1-fwd: GTCGGAGTGCGGAGACATG, prox1-rev: 5′-GGC CTT TTT CAA GTG ATT TGG A-3′, VEGFR-3-fwd: 5′-CCC CAG CCC TCA TTC CA-3′, VEGFR-3-rev: 5′-GCT GGG ACT GAC GA TAT TTG C-3′, lyve-1-fwd: 5′-CAT TCT GTG GCT CAA GGT GTC ATT AC-3′, lyve-1-rev: 5′-GCA TTT CTC ATT AGG CTG GAT ACG AG-3′, reelin-fwd: 5′-TAC AGT GGG TGG AAC CGA AT-3′, reelin-rev: 5′-GCT GGG CCA GAA AAT CCA GG-3′, ef1α-fwd: 5′-GAA CCA TCG AAA AGT TCG AGA AG-3′, ef1α-rev: 5′-TCC AAG ACC CAG GCA TAC TTG-3′.

### Morpholino injections

Fertilized *Xenopus* eggs from natural matings between hormone induced *Tg(Flk1:eGFP)* males and wild type females were injected at two-cell stage with xPROX1 (25 ng), xVEGF-C (35 ng) or standard control (35 ng) morpholino (Gene Tools, LLC, Philomath, OR). The ATG-targeted antisense morpholinos were designed based on published GenBank *Xenopus laevis* sequences of *xProx1* (#AB008773) and *xVEGF-C* (#CA973641) (Ny et al., 2005) and were as follows, xProx1: 5′-CAG GCA TCA CTG GAC TGT TAT TGT G-3′; xVEGF-C: 5′-GCT CCC TCC AGC AAG TAC ATT TTC C-3′; standard control morpholino: 5′-CCT CTT ACC TCA GTT ACA ATT TAT A-3′. Injected embryos were cultured in 0.1× MMR (Ny et al., 2005; Ny et al., 2008) at 18°C until gastrulation and thereafter at 22°C. Developmental stages of tadpoles were determined according to Nieuwkoop and Faber (Nieuwkoop and Faber, 1994). At stage 45 (5 days post fertilization (dpf)) tadpoles were subjected to lymphangiographies, angiographies and LEC labeling.

### Chemical compound treatment during development

At stage 26/28, *Tg(Flk1:eGFP)* tadpoles were placed in 6-well dishes (15–20 tadpoles/well) and up to 10 µM of MAZ51 (3-(4-dimethylamino-naphthelen-1-ylmethylene)-1,3-dihydroindol-2-one, Calbiochem–Merck Biosciences, Overijse, Belgium) was added to the tadpole growth media, as described previously (Ny et al., 2008). Control tadpoles were treated with the corresponding amount of DMSO. Compound/DMSO and growth medium were refreshed every day. At stage 45 (5 dpf), lymphangiographies and LEC labeling were performed to visualize the blood- and lymphatic vasculature.

### Pharmacological modulation of lymphatic sprouting in the fin

To visualize blood- and lymphatic sprouts from stage 47 onwards, *Tg(Flk1:eGFP)* tadpoles were LEC labeled as described above, 24 hours before the start of the experiment. MAZ51 (concentrations ranging from 1.25–5 µM) was added to LEC labeled tadpoles of stage 47 (placed in 10 cm petri dishes, with approximately 20 tadpoles per condition) in the tadpole growth media. Chemical compounds or buffer were refreshed daily. As a negative control tadpoles were treated with corresponding amounts of DMSO. After 7 days the number of lymphatic sprouts was counted over the entire length of the ventral axial vessels, and the cumulative length of the lymphatic sprouts was measured per tadpole with computer-assisted morphometry using the Zeiss KS300 software (Zeiss, Zaventem, Belgium) or the ImageJ software.

### Cell cluster-mediated modulation of fin sprouting

The *Xenopus laevis* kidney cell line A6 (CCL-102) was purchased from ATCC (LGC Standards, Teddington, UK). Cells were maintained in 75% NCTC 109 medium (Sigma–Aldrich, Bornem, Belgium), 15% distilled water (Invitrogen, Merelbeke, Belgium) and 10% fetal calf serum, supplemented with 2 mM glutamine, 100 U/ml Penicillin/Streptomycin, 0.01 M HEPES and 1 mM sodium pyruvate (all Invitrogen) at room temperature. Cells were passaged 1:3 twice a week. For transfection, cells were seeded at 70% confluency into 6-well plates and transfected using lipofectamine (Invitrogen, Merelbeke, Belgium) according to the manufacturer's instruction with plasmids coding for GFP, murine vascular endothelial growth factor (VEGF)-A or VEGF-C. Expression was verified by ELISA using Quantikine ELISA kits (R&D Systems, Abingdon, UK). Eighteen to 24 hours after transfection, cells were trypsinized, centrifuged and resuspended in a minimal amount of medium. Approximately 1,500 cells were injected as a dense cell suspension using a glass capillary, generating a local cell cluster within the two epithelial leaflets of the tadpole fin. Cells were never injected in one single injection, but were rather introduced using multiple low-pressure injections (typically 6–10) at the same site, thereby generating progressively larger cell clusters. The number of cells injected was controlled by verifying the diameter of the cell cluster. Only tadpoles with comparable cell cluster size were used in the experimental analysis. Sustained expression of the transgene by the injected cells was demonstrated by the green fluorescent signal of cells transfected with the GFP expression plasmid, persisting for a least 1 week. Injected tadpoles were allowed to recover from anesthesia and treatment with 2.5 µM MAZ51 was initiated by supplementation of the chemicals to the tadpole growth media. Buffers and chemicals were refreshed daily. After 4 days, the local effect on vessel sprouting was documented by pictures of the fin surrounding the central cell cluster. These pictures were taken at 50× magnification, representing approximately 1.4 mm on both sides of the cell cluster. The numbers of blood- and lymphatic sprouts were counted and the cumulative length of the sprouts was measured by computer-assisted morphometry using the Zeiss Axiovision KS300 software (Zeiss, Zaventem, Belgium) or the ImageJ software.

### Statistical analysis

All fin sprouting and cell injection experiments were analyzed using IBM SPSS Statistics 19. Data shown are estimated means and s.e.m. calculated from the combined results of at least 3 different experiments (except for the experiment using MAZ51 inhibition of VEGF-C overexpression effects; 2 experiments used). Estimated means, s.e.m. and significance levels were calculated with the general linear model multivariate statistical model, considering the number of blood vessels, number of lymph vessels, total blood vessel length and total lymph vessel length as dependent variables, compound dose and/or growth factor overexpressed as fixed factors, and experiment as co-variate. Pairwise comparisons were performed between the different doses, after Bonferroni correction for multiple testing. *P*<0.05 was considered statistically significant.

## Supplementary Material

Supplementary Material
