# Supplementary material for: A transgenic Xenopus laevis reporter model to study lymphangiogenesis
Source: Biol Open. 2013 Jul 11;2(9):882–90. doi: 10.1242/bio.20134739 (PMC3773334; doi:10.1242/bio.20134739)
Supplement: Supplementary Material [file supp_2_9_882__index.html]

A transgenic Xenopus laevis reporter model to study lymphangiogenesis — A transgenic Xenopus laevis reporter model to study lymphangiogenesis — Supplementary Material 

# A transgenic *Xenopus laevis* reporter model to study lymphangiogenesis

## 

**Files in this Data Supplement:**

- Supplementary Material - Annelii Ny et al. doi: 10.1242/bio.20134739
- Movie 1 - **Movie 1. Sprouting of lymph vessels in the ventral fin.** Time-lapse movie showing outgrowth of a lymphatic sprout with filopodia from the VCLV into the ventral fin. Arrow denotes the filopodia.
