# Supplementary material for: A transgenic Xenopus laevis reporter model to study lymphangiogenesis
Source: Biol Open. 2013 Jul 11;2(9):882–90. doi: 10.1242/bio.20134739 (PMC3773334; doi:10.1242/bio.20134739)
Supplement: Supplementary Material [file supp_bio.20134739_bio.20134739-s1.pdf]

Supplementary Material

Annelii Ny et al. doi: 10.1242/bio.20134739

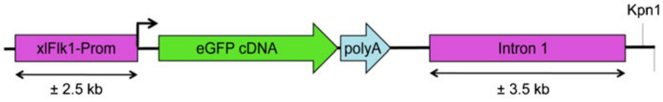

Fig. S1. The *Xenopus laevis Flk1:eGFP* reporter construct. Schematic overview of the *Xenopus laevis Flk1:eGFP* construct used to generate the *Tg(Flk1:eGFP)* transgenic line. The *eGFP* cDNA was placed under control of the *xlVEGFR-2/Flk1* upstream promotor sequence (approximately 2.5 kb) followed by the SV40 polyadenylation signal (polyA). Intron 1, containing the enhancer element essential for the correct expression pattern was cloned downstream of the *eGFP* cDNA and polyA. The construct was linearized using KpnI and used in restriction enzyme mediated integration (REMI) transgenesis.

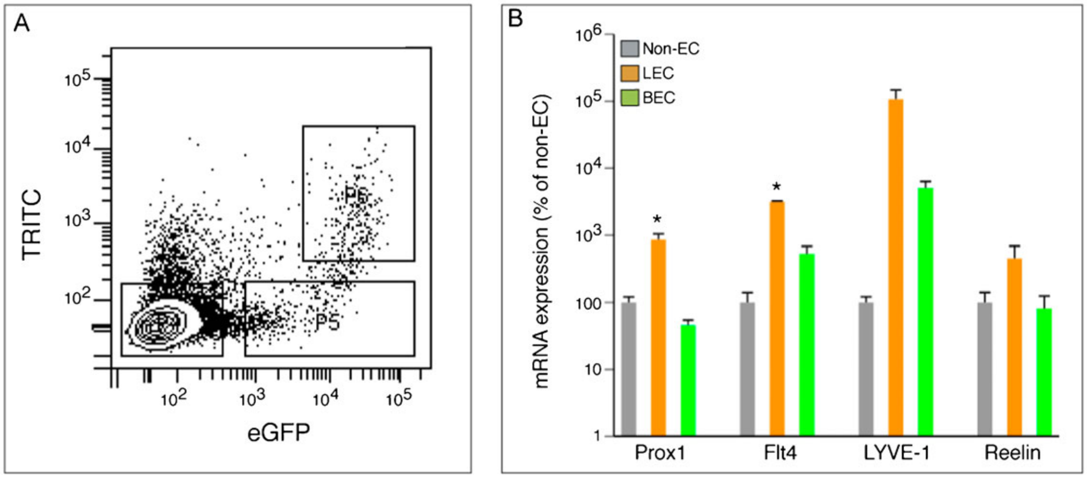

Fig. S2. FACS analysis of LEC-labeled tadpoles. (**A**) FACS dot plot showing the sorting set-up for isolating double TRITC$^+$eGFP$^+$ LECs (P6) and TRITC$^-$eGFP$^+$ BECs (P5) from stage 46 LEC-labeled tadpoles. LEC labeling was performed at stage 45, followed by homogenization of the tadpoles and isolation of the different EC populations 24 hours after labeling. (**B**) qRT-PCR on sorted BECs, LECs and eGFP$^-$ non-ECs (P4 in the FACS dot plot in panel A) showing significant enrichment for the LEC markers *Prox1* and *VEGFR-3* in the LEC population (orange bars) compared to the BEC population (green bars). For all genes mRNA expression is expressed relative to BEC expression. (*P<0.05 vs non-ECs or BECs). ND, not detectable. Quantitative data are mean ± s.e.m.

Biology Open

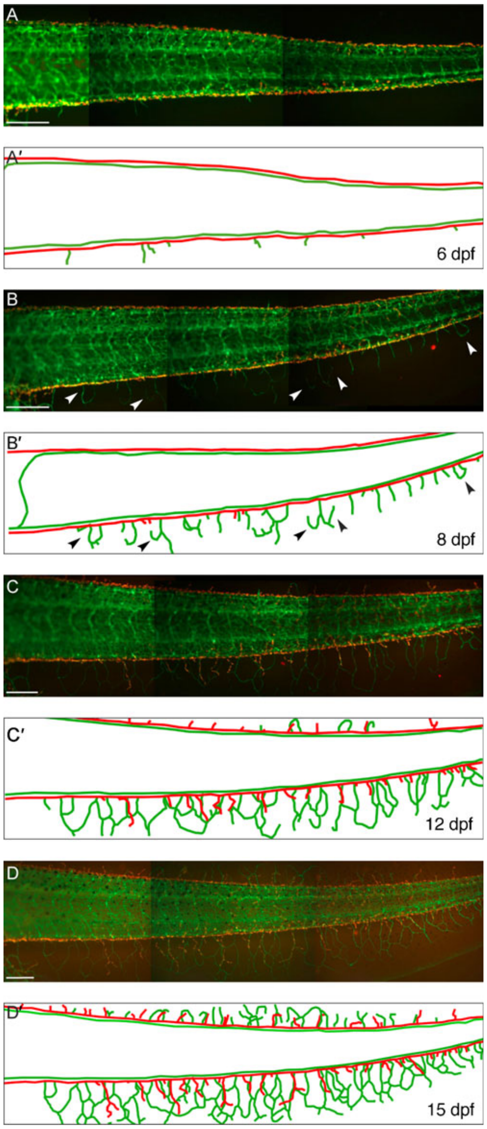

Fig. S3. Development of the blood- and lymphatic vascular network in the fin. All panels depict lateral views of the tadpoles, head facing left. Each fluorescence picture (**A–D**) is accompanied by a schematic redrawing of the blood vascular sprouts (green) and lymphatic sprouts (red) (**A′–D′**). A single tadpole was followed during the indicated stages. (A,A′) First blood vessel sprouting into the ventral fin is seen from stage 46 (6 days post fertilization (dpf)) onwards. (B,B′) Blood vessel sprouting is followed by lymphatic sprouting into the ventral fin, commencing at stage 47. Denoted by arrowheads, already formed blood vessels turning back towards the PCV to form a closed loop (8 dpf). (C,C′) Start of vessel sprouting into the dorsal fin (12 dpf). (D,D′) No or very few new blood and lymphatic sprouts are formed from the longitudinal vessels from this stage onwards (15 dpf and older). Instead, expansion of the dorsal and ventral vascular network depends on the growth of the already established primary sprouts and the formation of new secondary sprouts. Scale bars: 0.5 mm.

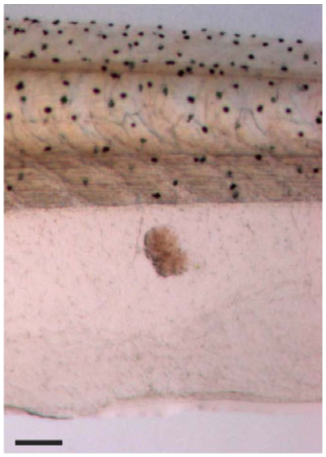

Fig. S4. Cell cluster formation after cell injection into the fin. Example of an injection of cells into the fin showing the formation of a cell cluster at the injection site. Injected cells remain clearly visible under the microscope for up to 1 week. Only tadpoles with comparable cell cluster size were used in the experimental analysis. Scale bar: 200 µm.

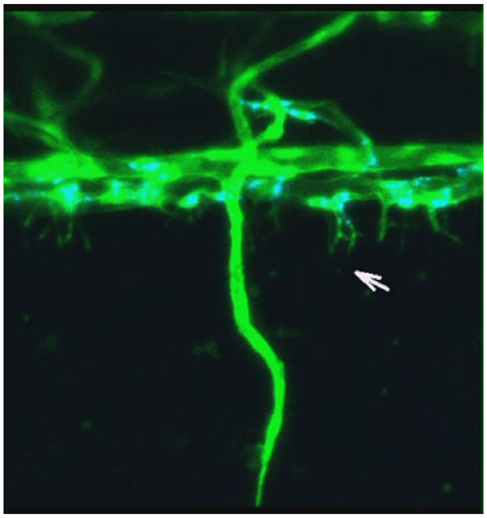

Movie 1. Sprouting of lymph vessels in the ventral fin. Time-lapse movie showing outgrowth of a lymphatic sprout with filopodia from the VCLV into the ventral fin. Arrow denotes the filopodia.

Table S1. Expression pattern and percentage germline transmission of *Tg(Flk1:eGFP)* founder lines.

Founder	Tadpoles screened (%GFP^+ germline offspring)	Expression pattern
1	1,361 (51%)	Heart, spinal cord, brain and vasculature (weak)
2	4,831 (51%)	Vasculature
3	1,491 (3%)	Vasculature
4	656 (51%)	Vasculature
5	363 (44%)	Brain
6	1,056 (0%)	–
7	833 (47%)	Spinal cord, brain and vasculature (weak)
8	1,780 (75%)	Vasculature
9	1,948 (52%)	Vasculature

Biology Open
